# What is Prevalence of Autism Spectrum Disorders in Republic of Georgia?

**DOI:** 10.1192/j.eurpsy.2022.600

**Published:** 2022-09-01

**Authors:** M. Zirakashvili, M. Gabunia, N. Mebonia, T. Mikiashvili, G. Chvamania, E. Kurashvili, V. Nadareishvili, S. Bishop, Y.S. Kim, B. Leventhal

**Affiliations:** 1 Ilia State University, Georgian Academy of Childhood Disability, Child And Adolescent Mental Health, Tbilisi, Georgia; 2 Georgian Academy of Childhood Disability, Child And Adolescent Mental Health, Tbilisi, Georgia; 3 Tbilisi State Medical University, Epidemiology, Tbilisi, Georgia; 4 Ilia State University, Child And Adolescent Mental Health, Tbilisi, Georgia; 5 University of California, San Francisco, Psychiatry And Behavioral Sciences Weill Institute For Neurosciences, San Francisco, United States of America

**Keywords:** autism, prevalence, Georgia

## Abstract

**Introduction:**

Despite the fact that 95% of all <5 years of age children with developmental disabilities including Autism Spectrum Disorders (ASD) live in low- and middle-income countries (LAMI), to date there is an information gap in LAMI studies including Republic of Georgia.

**Objectives:**

To estimate the prevalence and describe the clinical characteristics of ASDs among the third-grade school students using a total population approach.

**Methods:**

The target population (N=27,336) included all 3rd grade students of schools of five main cities of Georgia. The study was conducted in four steps: phase I screening, sampling of screen positive students, phase II confirmative diagnostic assessment, and best-estimate diagnosis. Parents and teachers completed two screening questionnaires in phase I: 27-item Autism Spectrum Screening Questionnaire (ASSQ) and 25-item Strengths and Difficulties Questionnaire (SDQ). In phase II, screen-positive children were evaluated using standardized diagnostic assessments.

**Results:**

Overall, 16,654 students (82%) were assessed by either parent and/or teacher. Students whose ASSQ and/or SDQ scores were in the top 10^th^ percentile were considered as screened positive for diagnostic assessment (N=1976). Of 300 students completed diagnostic assessment 53 were diagnosed ASD. Crude prevalence of ASDs was estimated to be 4.5%. 75% of cases of ASD were first diagnosed. Efforts are currently underway to compute adjusted prevalence, which will be available for the Conference presentation.

**Conclusions:**

The prevalence data of ASD is important to assess the burden of the disorder and facilitate better understanding of specifics of the disorder in different part of the world.

**Disclosure:**

No significant relationships.

